# An Immunocytochemistry Method to Investigate the Translationally Active HIV Reservoir

**DOI:** 10.3390/ijms26020682

**Published:** 2025-01-15

**Authors:** Guoxin Wu, Samuel H. Keller, Ryan T. Walters, Yuan Li, Jan Kristoff, Brian C. Magliaro, Paul Zuck, Tracy L. Diamond, Jill W. Maxwell, Carol Cheney, Qian Huang, Carl J. Balibar, Thomas Rush, Bonnie J. Howell, Luca Sardo

**Affiliations:** 1 MRL, Merck & Co., Inc., Rahway, NJ 07065, USA; 2 Pyramid Consulting, Inc., Alpharetta, GA 30022, USA

**Keywords:** immunocytochemistry (ICC), HIV, capsid (CA), CA-ICC, SIV, p27, qPCR, Simoa, flow cytometry, TACK

## Abstract

Despite the success of combination antiretroviral therapy (cART) to suppress HIV replication, HIV persists in a long-lived reservoir that can give rise to rebounding viremia upon cART cessation. The translationally active reservoir consists of HIV-infected cells that continue to produce viral proteins even in the presence of cART. These active reservoir cells are implicated in the resultant viremia upon cART cessation and likely contribute to chronic immune activation in people living with HIV (PLWH) on cART. Methodologies to quantify the active reservoir are needed. Here, an automated immunocytochemistry (ICC) assay coupled with computational image analysis to detect and quantify intracellular Gag capsid protein (CA) is described (CA-ICC). For this purpose, fixed cells were deposited on microscopy slides by the cytospin technique and stained with antibodies against CA by an automated stainer, followed by slide digitization. Nuclear staining was used to count the number of cells in the specimen, and the chromogenic signal was quantified to determine the percentage of CA-positive cells. In comparative analyses, digital ELISA, qPCR, and flow cytometry were used to validate CA-ICC. The specificity and sensitivity of CA-ICC were assessed by staining a cell line that expresses CA (MOLT IIIB) alongside a control cell line (Jurkat) devoid of this marker, as well as peripheral blood mononuclear cells (PBMCs) from HIV seronegative donors before or after ex vivo infection with an HIV laboratory strain. The sensitivity of CA-ICC was further assayed by spiking MOLT IIIB cells into uninfected Jurkat cells in limiting dilutions. In those analyses, CA-ICC could detect down to 10 CA-positive cells per million with a sensitivity superior to flow cytometry. To demonstrate the application of CA-ICC in pre-clinical research, bulk PBMCs obtained from mouse and non-human primate animal models were stained to detect HIV CA and SIV p27, respectively. The level of intracellular CA quantified by CA-ICC in PBMCs obtained from animal models was associated with plasma viral loads and cell-associated CA measured by qPCR and ELISA, respectively. The application of CA-ICC to evaluate the activity of small-molecule targeted activator of cell-kill (TACK) in clinical specimens is presented. Overall, CA-ICC offers a simple imaging method for specific and sensitive detection of CA-positive cells in bulk cell preparations.

## 1. Introduction

Progress has been made in understanding the molecular pathogenesis of HIV and has since led to the development of combination antiretroviral therapy (cART). However, cART only suppresses viral replication and cell-to-cell transmission, and its cessation is inevitably followed by viral rebound due to the existence of an HIV reservoir harboring HIV DNA [1,2,3,4]. The development of molecular tools that accurately measure the HIV reservoir size is critical to monitor remission and prognosis of HIV pathogenesis [5,6,7]. Currently, several approaches to quantify markers of the HIV reservoir exist, including those that measure the proviral DNA by PCR, the intact proviral DNA by digital PCR (IPDA), the HIV viral RNA by qPCR, the HIV capsid protein (CA) by digital single-molecule assay (Simoa), and the amount of replication-competent virus by quantitative viral outgrowth assay (QVOA) [6,8,9,10,11]. The value of these assays is limited by the HIV mutation rate and genomic deletions that render the majority of proviruses defective and unable to replicate [12]. Moreover, commonly used methods, such as the gold standard assay, QVOA, are expensive, labor-intensive, require a large volume of blood, and tend to underestimate the size of the replication-competent reservoir [9]. This highlights the need for simpler, less expensive, and time-saving methods to reliably measure the HIV reservoir size.

Immunocytochemistry (ICC) is a technique used to assess the presence of a specific protein or antigen in cells by use of an antibody to which it binds [13,14]. Here, bulk cells from cell lines or isolated from blood were used to quantify HIV CA-containing cells using an automated staining protocol coupled with computational image analysis. Specificity and sensitivity were assayed with cell lines and human PBMCs and compared to assays validated for quantifying viral biomarkers. Application of this method to the detection of CA-expressing cells in humanized mouse and non-human primate models and to the quantification of targeted activator of cell kill (TACK) activity [15,16] was also assessed. This novel application of ICC, named here CA-ICC, has the potential to provide a simple and sensitive alternative assay to quantify the translationally active HIV reservoir.

## 2. Results

### 2.1. CA-ICC Development and Characterization

**Figure 1:**
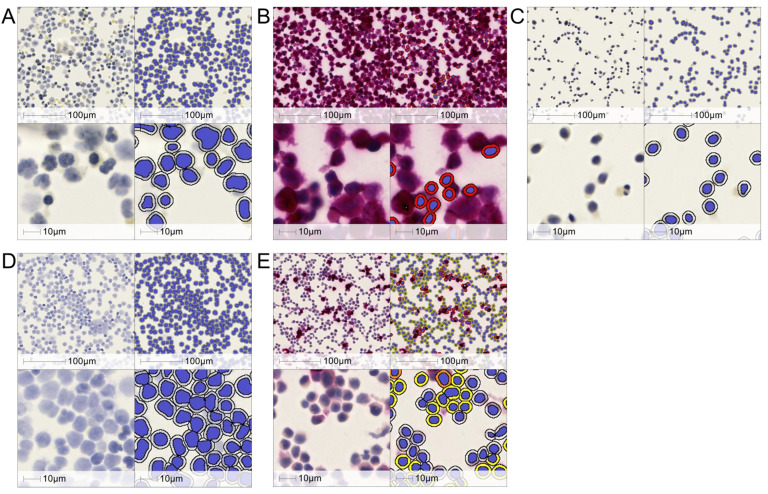
Sensitivity and specificity of CA-ICC. (**A**) MOLT IIIB cells stained with mIgG or (**B**) anti-CA antibodies. (**C**) Jurkat cells stained with anti-CA antibodies. (**D**) Uninfected and (**E**) in vitro infected human PBMCs stained with anti-CA antibodies. Representative raw images (**left panels**) and relative computational markup (**right panels**) are shown. Markup colors denote cells with strong (red), moderate (orange), and weak (yellow) chromogenic signal intensity. The top and bottom images for each panel represent 20× and 80× magnification, respectively.

To detect and quantify the number of HIV CA-containing cells, an immunocytochemistry assay (CA-ICC) was developed with two mouse anti-CA-specific antibodies at 3 µg/mL each. These two monoclonal antibodies (mAbs) recognize different regions of CA, whereby the binding domain of the first anti-CA mAb (ZeptoMetrix, Buffalo, NY, USA) is located at the C-terminus of CA and the second anti-CA mAb (Capricorn) is located in the middle region of CA (Table 1 and Section 4.1). To improve detection sensitivity and specificity, both anti-CA mAbs were used together in this study. The sensitivity of CA-ICC was first evaluated with MOLT IIIB cells, a human T-cell line chronically infected with HIV that constitutively produces HIV proteins in the cell and virus particles in the culture supernatant [17], and with in vitro infected human PBMCs (Figure 1). As a negative technical control, MOLT IIIB cells were stained with a mouse IgG (mIgG) that does not bind to HIV-CA, demonstrating that none of the cells with positive nuclear staining displayed the CA signal, as expected (Figure 1A and Table 1). To the contrary, staining of MOLT IIIB with anti-CA antibodies displayed the intracellular chromogenic signal for 99.9% of cells with positive nuclear staining, as expected (Figure 1B and Table 1). Expressed as a probability, the CA-ICC’s sensitivity, calculated as the ratio between the number of true positives and the sum of the number of true positives and false negatives [26,034/(26,034 + 19) × 100], was 99.96%. As negative biological controls, Jurkat cells (which do not express CA) and PBMCs from HIV seronegative individuals did not display any intracellular capsid signals when stained with anti-CA antibodies (Figure 1C,D and Table 1). Expressed as a probability, the CA-ICC’s specificity, calculated as the ratio between the number of true negatives and the sum of the number of true negatives and false positives [69,410/(69,410 + 0) × 100], was 100%. Under these conditions, staining of in vitro HIV-infected PBMCs displayed 73.3% cells with the CA intracellular signal (Figure 1E and Table 1). All together, these data showed that CA-ICC is sensitive and specific for the detection of intracellular CA in cell lines and in primary cells infected in vitro.

**Figure 2:**
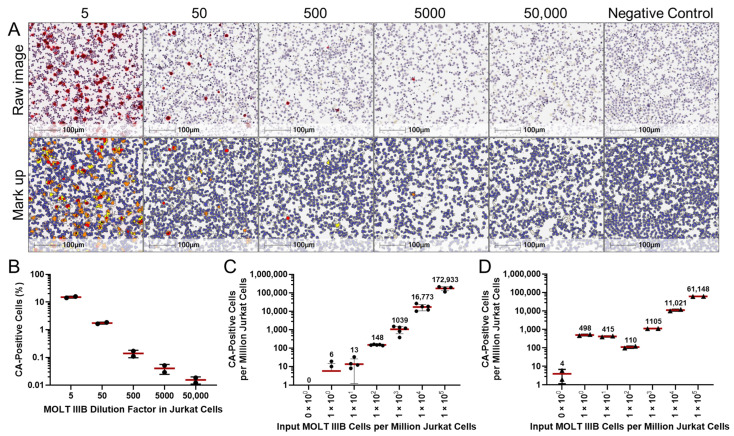
CA-ICC sensitivity and comparison with flow cytometry. (**A**) Representative raw images with relative computational markup of MOLT IIIB spiked into Jurkat cells with serial dilutions and (**B**) relative frequency of HIV CA-positively stained cells by CA-ICC. Markup colors denote cells with strong (red), moderate (orange), and weak (yellow) chromogenic signal intensity. Comparative analysis of the limit of detection of (**C**) CA-ICC and (**D**) flow cytometry, and accuracy of the recovery of CA-positive MOLT IIIB cells spiked into uninfected Jurkat cells. The values above the dot plots represent observed frequencies.

Additional experimentation was performed to assess CA-ICC’s sensitivity (Figure 2). For this purpose, chronically infected MOLT IIIB cells were spiked into Jurkat cells starting at 1:5 dilution (200,000 MOLT IIIB cells into 800,000 Jurkat cells) and then at 1:10 serial dilutions up to 1:50,000 (16 MOLT IIIB cells into 800,000 Jurkat cells). On average, 95,293 ± 27,388 cells were scanned per serial dilution (Figure 2B). The percentage of CA-positive cells detected by CA-ICC decreased progressively with increasing dilutions, showing frequencies close to the expected values. Specifically, the detection percentages decreased from 15% at the 1:5 dilution to 1.7% at the 1:50 dilution, 0.14% at the 1:500 dilution, 0.04% at the 1:5000 dilution, and 0.015% at the 1:50,000 dilution (Figure 2A,B). To validate CA-ICC, HIV CA and RNA markers were quantified by standard ELISA and qPCR assays, respectively, using cell lysates from the same set of MOLT IIIB cells diluted in Jurkat cells. The concentrations of CA measured as picograms per milliliter were 48.959, 4.167, 0.543, 0.022, and 0.006 in the 1:5, 1:50, 1:500, 1:5000, and 1:50,000 dilutions, respectively. Independently, the numbers of HIV-RNA copies per microliter were 1285.4, 124.5, 12.1, 1, and 0.3, respectively. The proportion of CA-positive cells measured by CA-ICC was associated with the quantification of CA in cell lysate by ELISA and with the intracellular viral RNA measured by qPCR. These data indicated that the quantifications made with CA-ICC were consistent with other known biomarkers of intracellular HIV replication, including CA and viral RNA measured with standard assays. Separately, CA-ICC’s reproducibility was evaluated by spiking MOLT IIIB cells into Jurkat cells at 7% dilution. On average, 165,798 ± 12,527 cells were scanned per condition. As shown in Table 2, the coefficient of variations (CVs) of intra-day and inter-day were 12.97% (N = 3) and 10.08% (N = 3), respectively. Both CVs were below 20%, indicating that CA-ICC was reproducible when performed three times within the same day or when performed on three consecutive days (Table 2).

To compare CA-ICC’s sensitivity with validated flow cytometric detection of CA, MOLT IIIB cells were spiked into Jurkat cells at different dilutions to obtain expected frequencies ranging from 1 to 100,000 CA-positive cells per million (Figure 2C,D). As a negative control, Jurkat cells were used to set the baseline (Figure 2C,D; ‘0’ cells condition). These samples were then split and analyzed by CA-ICC and flow cytometry in parallel to compare the sensitivity of the two assays. On average, 194,581 ± 40,005 cells per dilution were scanned for CA-ICC analysis. By CA-ICC, no CA-positive cells were detected in the sample containing only Jurkat cells. On average, 6, 13, 148, 1039, 16,773, and 172,933 CA-positive cells were observed in the samples with expected frequencies of 1, 10, 100, 1000, 10,000, and 100,000 per million, respectively. It should be noted that, in these experiments, normalization per million was achieved by extrapolation. With the exception of the condition testing the 1 cell per million frequency, which returned an average value 6-fold greater than expected, CA-ICC’s recovery rates were consistently within 2-fold of the expected frequencies and displayed a linear range throughout the serial dilutions (Figure 2C). On the other hand, the average numbers of CA-positive cells observed by flow cytometry in the serial dilutions described above were 498, 415, 110, 1105, 11,021, and 61,148, respectively. In this case, the dilutions equal to and greater than 1000 CA-positive cells per million observed recovery rates within 2-fold of the expected frequencies and a serial linear range. However, the average number of CA-positive cells observed in the dilutions containing 100 or fewer CA-positive cells per million was inconsistent with the expected frequencies. On average, 4 positive cells per million were quantified in the sample where the expected frequency was 0. These discrepancies highlighted the limitations of flow cytometry for the quantification of these samples (Figure 2D). Taken together, CA-ICC was demonstrated to be specific, reproducible, and to possess a linear range for the detection of CA-positive cells at expected analyte concentrations. In this regard, CA-ICC was shown to be more specific and sensitive than standard flow cytometric detection of CA.

### 2.2. CA-ICC of Bulk PBMCs Isolated from Blood Obtained from Animal Models

**Figure 3:**
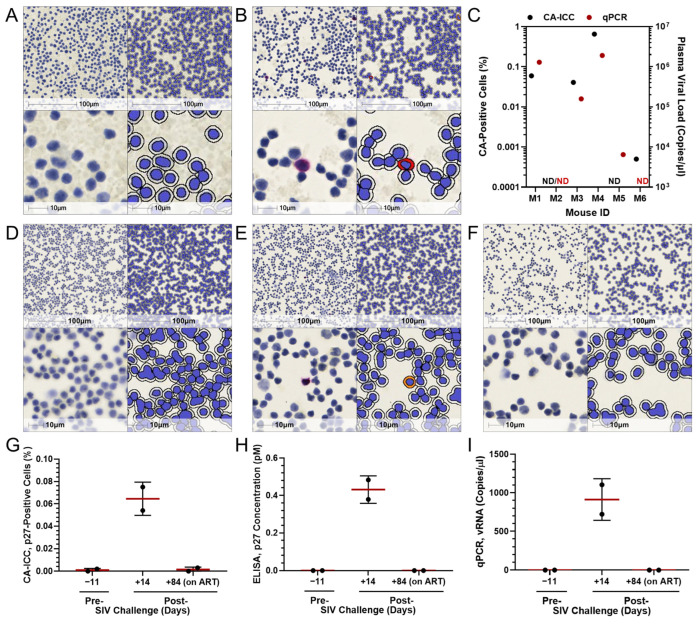
Application of CA-ICC to PBMCs derived from animal models. CA-ICC mediated detection of HIV-CA in cells obtained by retro-orbital bleeding of (**A**) uninfected and (**B**) viremic mice. (**C**) Association between the proportion of CA-positive cells detected by CA-ICC and plasma viral loads (ND: not detected). CA-ICC mediated detection of SIV p27 in rhesus macaques’ PBMCs (**D**) prior to challenge, (**E**) 14 days, and (**F**) 84 days post-challenge (cART initiated at day 56). (**G**) Quantification of cell-associated SIV p27 by CA-ICC, **(H**) ELISA, and (**I**) cell-associated SIV-RNA by qPCR. Representative raw images and relative computational markup are shown. Markup colors denote cells with strong (red), moderate (orange), and weak (yellow) chromogenic signal intensity. The top and bottom panels represent 20× and 80× magnification, respectively.

As an application of CA-ICC for the detection of in vivo biomarkers, blood samples collected from a humanized mouse model (Figure 3A–C) and a non-human primate model (Figure 3D–I) were used. Cells expressing HIV-CA intracellularly were detected in blood samples collected from mice engrafted with in vitro HIV-infected human PBMCs (Figure 3B) but not in control animals (Figure 3A). On average, 231,197 ± 82,830 PBMCs were scanned by CA-ICC for each of the six mice included in the study. Due to the limited number of cells obtainable from retro-orbital bleeding in mice, CA-ICC quantification was compared to plasma viral loads. This comparison revealed a range of viremia set points and demonstrated an association between the two markers (Figure 3C).

To evaluate whether CA-ICC could also be used for the detection of SIV p27, a mouse anti-p27-specific mAb was used in addition to an anti-CA antibody that cross binds with both HIV CA and SIV p27. Staining of in vitro SIV-infected rhesus macaque’s PBMCs displayed ~27% cells with the detectable p27 signal by CA-ICC. As a negative control, the same sample was stained with mIgG and did not display any positive cells, suggesting that the p27 signal was specific. To further test the applicability of CA-ICC to a non-human primate model, PBMCs longitudinally isolated from two individuals before SIV infection (day −11), after SIV infection (day 14), and after initiation of antiretroviral treatment (day 84) were evaluated. On average, 226,776 ± 106,614 PBMCs were scanned for each of the two monkeys included in the study (Figure 3D–F). The percentages of p27-positive cells at day −11, day 14, and day 84 were 0.001 ± 0.002, 0.065 ± 0.015, and 0.001 ± 0.002, respectively (Figure 3G). As a comparison, p27 accumulation in PBMCs lysate (Figure 3H) and plasma viral loads (Figure 3I) were measured in the same blood samples with Simoa-based and qPCR validated assays, respectively. The readouts from the different assays displayed comparable trends across the time course (Figure 3G–I). Altogether, the datasets obtained from the two animal models showed that CA-ICC can be used to determine the proportion of CA-expressing cells and that its quantification was associated with other biomarkers of virus replication assessed with previously validated assays.

### 2.3. Measurement of CA-Expressing Cells in HIV-Infected Human PBMCs Treated with Antiretrovirals

**Figure 4:**
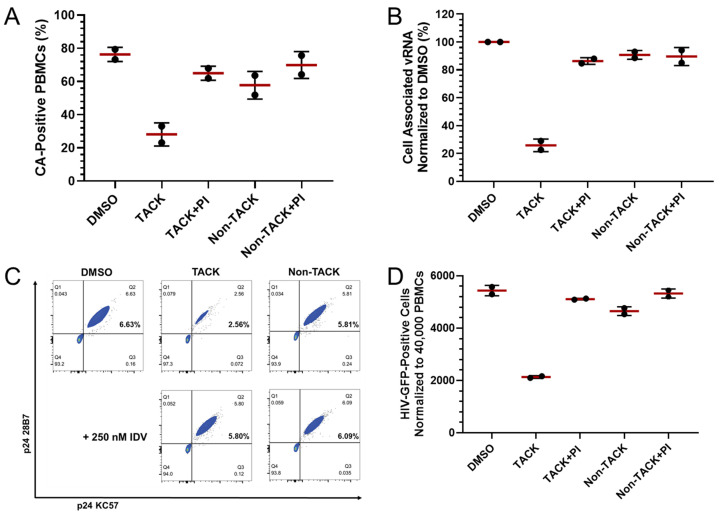
Application of CA-ICC to the quantification of the activity of TACK. (**A**) Quantification of CA-positive cells by CA-ICC. (**B**) Quantification of cell-associated viral RNA by qPCR. (**C**) Quantification of CA-positive CD4+ T cells by flow cytometry. (**D**) Quantification of GFP-positive cells by microplate reader. Non-TACK: TACK inactive NNRTI; IDV: HIV protease inhibitor indinavir; PI: protease inhibitor.

To evaluate applications of CA-ICC in drug discovery and development, CA-ICC was used to assess the activity of small-molecule targeted activator of cell kill (TACK) [16]. TACK compounds are non-nucleoside reverse transcriptase inhibitors (NNRTIs) that can induce HIV-selective cytotoxicity through binding to the RT domain of monomeric Gag-Pol and causing premature intracellular viral protease activation. For this purpose, human PBMCs were infected with a replication-incompetent virus expressing a GFP reporter. Twenty-four hours post-infection, the cells were treated with compound for 72 h. Viral biomarkers were quantified by CA-ICC and validated using qPCR-, flow cytometric- and GFP-based assays (Figure 4). As negative controls, an NNRTI that does not possess TACK activity (non-TACK in Figure 4) and a co-treatment consisting of TACK along with the HIV protease inhibitor (PI) indinavir (IDV), which is known to block TACK activity, were used. For CA-ICC quantification, an average of 187,798 ± 23,086 cells were scanned across all treatments. The proportion of HIV-positive cells detected by CA-ICC was 76.3% following HIV infection and treatment with vehicle control (DMSO) (Figure 4A). Treatment with TACK reduced the number of CA-positive cells by 2.7-fold to 28.1%, while this effect was blocked by the addition of IDV. As expected, the non-TACK compound treatment displayed proportions of CA-positive cells comparable to the vehicle control (Figure 4A). To evaluate the hypothesis that the amount of intracellular CA signal is associated with susceptibility to TACK-mediated elimination, CA-positive cells were sorted based on the intensity of the chromogenic signal quantified by computational analysis. In the experiment presented in Figure 4A, three subgroups of CA-positive cells were arbitrarily defined as strong, moderate, and weak, accounting for average percentages of 8.6, 12.9, and 54.7 of the total CA-positive cells (~76% as described above), respectively. The average percentages of CA-positive cells quantified after TACK treatment in the three subgroups were 2.3, 4.0, and 21.9 of the total CA-positive cells (~28%, as described above), respectively. The reductions in CA-positive cells in the strong, moderate, and weak subgroups were 3.8-, 3.2-, and 2.5-fold, respectively, suggesting that cells with a higher intensity of intracellular CA (and possibly of Gag-Pol) signal were more susceptible to TACK-mediated cell elimination.

The same samples, treated under the conditions described above, were used to quantify other markers of virus replication using standard assays. TACK treatment lowered the level of viral RNA-positive cells detected by qPCR by 3.9-fold (Figure 4B), CA-positive cells detected by flow cytometry by 2.6-fold (Figure 4C), and the number of GFP-positive cells detected by a microplate reader by 2.5-fold (Figure 4D), with these last two protein markers’ reductions being comparable to CA-ICC quantification. The data indicated that CA-ICC could quantify the activity of antiretroviral agents that specifically eliminate cells expressing CA, similarly to standard methods.

**Figure 5:**
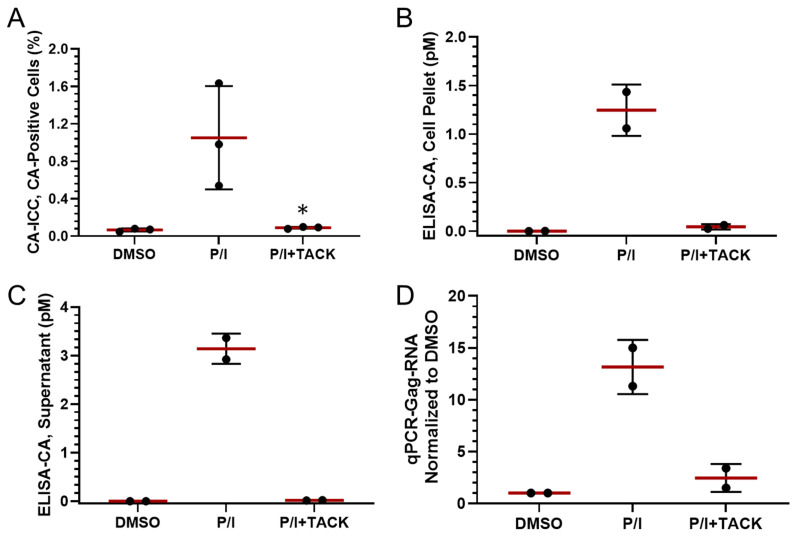
Validation of CA-ICC with clinical specimens after ex vivo treatment with TACK. (**A**) Quantification of CA-positive cells by CA-ICC. Quantification of CA by ELISA in (**B**) cell pellets and (**C**) culture supernatants. (**D**) Quantification of cell-associated HIV-RNA by qPCR. Statistical significance in group comparisons was denoted conventionally by *, *p* < 0.05 using the Student’s *t* test.

Finally, to evaluate the use of CA-ICC with clinical samples, bulk PBMCs from three PLWHs on cART were stimulated with PMA/Ionomycin (P/I) to reactivate cells from latency and treated with TACK to eliminate CA-expressing cells (Figure 5). In those experiments, approximately 100,000 cells were scanned per donor per condition, and less than 0.1% CA-positive cells were detected by CA-ICC in the vehicle control treatment (DMSO), while P/I stimulation increased the proportion of CA-positive cells to ~1% (Figure 5A). Treatment of P/I-reactivated cells with TACK reduced the proportion of capsid expressing cells to ~0.1% (*p* < 0.05). As confirmatory evaluation using a standard assay, CA measured by Simoa showed similar treatment-induced patterns in both the cell lysate (Figure 5B) and culture supernatant (Figure 5C). In addition, the efficacy of TACK treatment, assessed by HIV viral genomic RNA quantification using qPCR, was comparable to that assessed by CA-ICC (Figure 5D). Altogether, this validation indicated that CA-ICC can be used to measure the pharmacological reduction of translationally active HIV-infected cells in clinical samples as an alternative to standard assays.

## 3. Discussion

Currently, several assays to quantify viral reservoir markers in bulk cells are available, including those that measure the HIV provirus (DNA), viral genomic RNA, and viral proteins [8,18,19]. Traditional assays aimed at reservoir characterization by measuring HIV DNA or RNA are sensitive and scalable but have limitations in distinguishing defective from translation- and replication-competent proviruses [7,20]. This can inherently lead to an overestimation of the size of the reservoir. In addition, standard cell culture-based assays, such as QVOA, that measure HIV Gag CA in culture medium after expansion in culture for 2–3 weeks, require large sample volumes and have limited throughput and clinical application [9]. It should also be emphasized that the successful translation of Gag does not unequivocally confirm the generation of replication-competent progeny viral particles. Alternatively, the Tat/rev-induced limiting dilution assay (TILDA), which measures cell-associated multiply spliced HIV RNA, offers advantages to QVOA, such as increased throughput and decreased sample requirements, but only measures RNA transcripts [8]. Overall, these assays require cells lysis as end point quantification and preclude analysis of cell morphology and visualization of subcellular biomarkers’ localization. Thus, alternative approaches to quantitate the translationally active reservoir in bulk cells are needed to directly visualize CA at the single cell level.

In this work, a novel immunocytochemistry method (CA-ICC), employing antibody-mediated detection of intracellular HIV CA in combination with automated staining and computational image analysis, is described. CA-ICC was validated with HIV-infected cell lines and primary cells, and its application was tested with blood samples collected from animal models. Across multiple experiments, the quantification of CA-positive cells made by CA-ICC was associated with other viral markers quantified with validated methods (Figure 2, Figure 3, Figure 4 and Figure 5). Importantly, when compared to standard flow cytometric detection of CA, CA-ICC displayed improved specificity and sensitivity in quantifying CA-positive cells spiked into uninfected Jurkat cells (Figure 2C,D). In those experiments, CA-ICC allowed detection within a 2-fold range from expected frequencies, with an improved linear range resolution down to 10 CA-positive cells per million. However, in samples with an expected frequency of 1 CA-positive cell per million, the average observed quantification by CA-ICC was 6-fold larger than expected. This indicates that while CA-ICC shows promise, further optimization is needed to improve its sensitivity at such low frequencies (Figure 2C). Nonetheless, within the same samples, the flow cytometric quantification of CA-positive cells at frequencies below 100 per million was inaccurate, and the background signal posed limitations (Figure 2D). Thus, CA-ICC is superior to flow cytometric quantification of intracellular CA, even though the mAbs used for CA-ICC and flow cytometry, as well as the respective methodologies, are different. Overall, this improvement offers a clear advantage of using CA-ICC over flow cytometry for the detection of the translationally active reservoir. It should be noted that while the FISH-Flow assay allows for the detection of about 1 Gag-positive cell per million and it appears to be more sensitive than CA-ICC in that regard, it lacks a microscopic visualization of CA in the cell and requires a more laborious procedure than CA-ICC [21]. In this regard, CA-ICC is an advantageous method because it uses a relatively simpler protocol based on commercially available reagents and instrumentation. Compared to QVOA, another methodology for the detection of the translationally active reservoir, CA-ICC is favorable as its protocol is two days long, as opposed to several weeks. Furthermore, CA-ICC requires smaller blood volumes than QVOA, which is critical for preserving precious clinical samples. Importantly, one autostainer can stain 30 slides in one run, and two experiments can be performed per day, thereby expanding the throughput of CA-ICC to 60 samples per day. As a disadvantage, the CA-ICC’s capacity, as determined by the number of cells that can be loaded on a microscopy slide, is often limited to 0.5 million cells per specimen. Thus, samples with larger numbers of cells need to be separately loaded onto multiple slides. However, using two loading zones within the same microscopy slide is an adjustment currently under evaluation.

Among the factors contributing to the sensitivity of CA-ICC is the use of a combination of two mAbs that bind to different epitopes in CA to minimize the detection of false negatives. Furthermore, according to the manufacturer’s description, the BOND Polymer Refine Red detection kit employs a controlled polymerization technique to create polymeric AP-linker antibody conjugates. This method eliminates the need for traditional streptavidin and biotin processes, thus significantly reducing nonspecific staining. In addition, the commercially available automated staining system used for the CA-ICC procedure, as described in Section 4.2 and Section 4.3, is typically employed in digital pathology workflows designed to detect signals in tissue specimens. These specimens are inherently more challenging to stain than bulk cell preparations due to factors such as specimen fixation, dehydration, and permeabilization. It should be noted, however, that the requirement for specialized equipment, including an autostainer and digital pathology scanner, is a disadvantage of the CA-ICC protocol. Nonetheless, it is not more costly than flow cytometry.

To demonstrate the applicability to clinical samples, CA-ICC was used to quantify the activity of TACK to induce cell death of either in vitro infected PBMCs or ex vivo treated PBMCs from PLWHs on cART (Figure 4 and Figure 5). TACK compounds are antiretrovirals belonging to the NNRTI class that can trigger the premature activation of HIV protease followed by CARD8-mediated activation of the inflammasome and pyroptosis in the infected cell, which provides a novel mechanism to reduce the translationally active reservoir. CA-ICC quantification of TACK’s efficacy was comparable to previously validated assays based on quantitative PCR, flow cytometry, and GFP fluorescence, further showing that CA-ICC is a valid alternative to those methods.

Although at this stage CA-ICC is limited to detection of the intracellular CA marker and analyses were conducted with modest sample sizes, studies are ongoing to multiplex the simultaneous detection of additional markers and to apply this technique to cells obtained from dissociated tissue. Coupling the detection of CA with other viral and host markers, along with the ICC’s capability to visualize their subcellular distribution at the single cell level, will enhance the complexity of characterizing the translationally active reservoir. These additional applications should improve the potential of CA-ICC to become a simple and rapid tool to profile the translationally active reservoir in pre-clinical and clinical samples.

## 4. Materials and Methods

### 4.1. Antibodies and Cells

The antibodies to HIV CA or SIV p27 used in this study are listed in Table 3. The binding domain of the ZeptoMetrix anti-HIV CA mAb is located within the p24 amino acid position 46–75 (GATPQDLNTMLNTVGGHQAAMQMLKETINE), as determined by mass spectrometry. The Capricorn anti-HIV CA mAb binding domain is instead located at p24 amino acid position 188–197 (TLLVQNANPD), as determined by peptide mapping. When aligned with 11,677 complete HIV-1 sequences in the Los Alamos National Laboratory database (2021 version), the amino acid sequences of the binding sites for the ZeptoMetrix and Capricorn mAbs were conserved at rates of 31.9% and 87.2%, respectively. In addition, as shown in a previous report, the combination of these two mAbs efficiently detects p24 from multiple HIV-1 subtypes [22]. The sequence of the ABL anti-SIV p27 mAb binding domain is located at the p27 amino acid position 64–84 (AMQIIRDIINEEAADWDLQH), as determined by peptide mapping.

The MOLT IIIB cell line carrying an integrated HIV provirus was obtained from the AIDS Research Program [17]. Human PBMCs isolated from leukapheresis were obtained from Lonza (Basel, Switzerland). Jurkat cell line clone E6-1 was obtained from ATCC. Non-human primate (NHP) rhesus macaque PBMCs were isolated from whole ethylenediaminetetraacetic acid (EDTA) anticoagulant blood by Ficoll density gradient centrifugation. Mouse PBMCs were isolated from mouse whole blood, collected in an EDTA tube, spun down, and the cell pellet was treated with red blood cell lysis buffer (Biolegend, San Diego, CA, USA).

### 4.2. CA-ICC Slide Preparation and Workflow

Cell pellets from either cell lines or PBMCs were washed once with PBS and fixed with 4% paraformaldehyde (PFA) for 2 h at room temperature. Fixed cells were resuspended to a 1 × 10^6^ cells/mL density in PBS, and a 0.5 mL cell mixture (0.5 × 10^6^ cells) was loaded into a cytospin funnel cassette (Thermo-Fisher, Carlsbad, CA, USA) with a cytospin single slide (Thermo-Fisher). Cells were spun at 800 rpm for 10 min at room temperature (RT) in a CytoSpin 4 Cytocentrifuge. Slides were air dried for 30 min at RT and immersed in 50% ethanol (EtOH) for 5 min, followed by immersions in 70% EtOH for 5 min and 100% EtOH for 5 min. The slides were then stored in 100% EtOH for up to 1 month at −20 °C until staining.

### 4.3. CA-ICC

Slides were stained by a Leica Bond RX autostainer (Leica Biosystems, Nussloch, Germany) via a BOND Polymer Refine Red Detection kit (Leica Biosystems). For the detection of HIV-p24, two anti-CA mAbs (anti-CA HIV-018-48304 and anti-CA 801136; Table 3) were combined to increase the staining sensitivity. For the detection of SIV-p27, two antibodies (anti-SIV p27 4324 and anti-HIV CA HIV-018-48304; Table 3) were combined. Antibodies were diluted with Da Vinci Green Diluent buffer (Biocare Medical, Concord, CA, USA), each to final concentration of 3 µg/mL. A secondary alkaline phosphatase (AP)-labeled anti-mouse IgG detection antibody and AP red substrate were included in the kit. All of the staining procedures were performed according to the manufacturer’s instructions with an automated protocol. After staining, the slides were washed once with distilled water for 1 min, then dehydrated twice with 100% EtOH for 5 min, followed by two immersions in HistoPrep xylene (Fisherbrand, Pittsburgh, PA, USA), for 5 min each. The slides were mounted with one drop of EcoMount (Biocare Medical) and a coverslip, then dried overnight at RT. The prepared slides were scanned using a Zeiss Axioscan (Oberkochen, Germany) and, after acquisition, the images were analyzed and rendered with a digital pathology imaging software (HALO, v3.6.4134, Indica Labs, Albuquerque, NM, USA) [23].

### 4.4. Preparation of Mouse and NHP Bulk PBMCs

For CA-ICC validation, PBMCs were collected from two animal models, a mouse HIV model and a SIV-infected rhesus macaque model. The mouse HIV model utilized intraperitoneal (IP) injection of HIV-infected human PBMCs. For this, fresh human PBMCs were activated with phytohemagglutinin (PHA) at a concentration of 5 µg/mL in complete RPMI-1640 (cRPMI) medium with 10% fetal bovine serum (FBS) and 20 U/mL Interleukin-2 (IL-2) for 3 days. After activation, PBMCs were infected with the R8 wild-type replication-competent HIV laboratory strain [24] with a multiplicity of infection (MOI) of 0.1 for 4 h. Following infection, the input virus was washed away with cRPMI three times. The infected PBMCs were then cultured in T175 flasks with fresh cRPMI. Three days after infection, the cultured PBMCs were collected. A concentration of 30 × 10^6^ PBMCs/mL in phosphate-buffered saline (PBS) was injected into immunodeficient NOD-SCID-IL-2ry−/− mice. Mouse blood was collected by retro-orbital bleeding, both before and 5 weeks post-injection of HIV-infected human PBMCs, for both CA-ICC and plasma viral load analyses.

For the SIV-infected non-human primate model, PBMCs were isolated from rhesus macaques pre-infection (day −11), post infection (day 14) with SIVmac239m [25], and post-treatment (day 84; 28 days post-treatment initiation with daily subcutaneous 2.5 mg/kg dolutegravir, 40 mg/kg emtricitabine, 5.1 mg/kg tenofovir disoproxil fumarate) [26]. The isolated PBMCs were used for CA-ICC, p27 Simoa, and SIV viral RNA measurements.

In addition, for in vitro characterization of p27 CA-ICC, PBMCs were isolated from uninfected rhesus macaques’ blood, activated with 5 µg/mL Concanavalin A in cRPMI for 3 days, washed and resuspended in culture media with 20 U/mL IL-2. PBMCs at a ~9.6 × 10^6^/mL density were then infected with SIV SF162p3 in a 50 mL bio-reactor tube with spin inoculation at 2000× *g* for 2 h at RT. Infected PBMCs were then cultured overnight, washed three times with cRPMI by centrifuging at 200× *g* for 5 min, and fixed with 4% PFA for p27 CA-ICC.

### 4.5. Plasma Viral Load Measurement

Murine blood was collected by retro-orbital bleeding before and after injection of HIV-infected PBMCs. Plasma was obtained by centrifuging the blood at 2000× *g* for 5 min in an Eppendorf tube. Quantitative PCR (qPCR) was performed using a primers/probe set targeting the HIV integrase sequence, similarly to our previous procedure [23]. To determine the standard curve, HIV RNA was extracted from the MOLT IIIB cell line [17] and quantified using the QuantStudio 3D digital real-time PCR system (QuantStudio 3D Analysis Suite Cloud Software, 15 April 2016, Thermo Fisher Scientific, Carlsbad, CA, USA). The plasma HIV viral loads (copies/mL) were calculated based on the standard curve.

### 4.6. Single Molecule Array (Simoa)

Supernatant obtained from the cell culture was centrifuged at 10,000× *g* for 5 min at RT to remove insoluble material before measuring CA levels on the Quanterix analyzer (Quanterix, Bullerica, MA, USA). The assay reagents and reaction conditions for the CA measurements were previously described [22,27]. The concentration of CA was determined using a calibration curve fit to a four-parameter logistic model.

### 4.7. Quantitative Reverse-Transcription Polymerase Chain Reaction (qPCR)

PBMCs were isolated from PLWH on cART using either leukapheresis or whole blood via Ficoll-gradient centrifugation. CD4+ T cells were then isolated from PBMCs using negative selection with the EasySep Kit (STEMCELL Technologies, Vancouver, BC, Canada). CD4+ T cells were treated with either 0.1% DMSO or a combination of 10 ng/mL PMA (phorbol 12-myristate 13-acetate) and 1 μg/mL ionomycin in culture medium for 48 h. The cells and culture medium were collected by centrifugation, and RNA was isolated from the cell pellet using the RNeasy kit (Qiagen, Hilden, Germany). qPCR was performed using the TaqMan Fast Virus 1-Step Master Mix (Thermo Fisher) [23]. A total of 2 µL of purified RNA was used as template. Gene expression assays for the primers/probe specific to HIV or SIV gag [28] were obtained from Thermo Fisher. qPCR was carried out using the QuantStudio 12K Flex system (ThermoFisher Scientific, Carlsbad, CA, USA).

### 4.8. Flow Cytometry

PBMCs were stained as previously described [29], with modifications. Briefly, cells were stained for viability at 4 °C for 20 min using BD Horizon Fixable Viability Stain 700 (BD Biosciences, Franklin Lakes, NJ, USA) and blocked for 10 min at RT with Human TruStain FcX (Biolegend) prior to surface staining. CD4+ T cells were then stained for surface markers at 4 °C for 30 min with the following antibodies in PBS, including 1% FBS and Brilliant Stain Buffer Plus (BD Biosciences): CD3-BUV496 (UCHT-1, BD Biosciences), CD4-BV786 (SK3, BD Biosciences), and CD8-BUV737 (SK1, BD Biosciences). Fixation and permeabilization of cells were performed at 4 °C for 20 min using BD Cytofix/Cytoperm Buffer (BD Biosciences), followed by intracellular staining for 45 min at RT with anti-CA-KC57-PE and anti-CA 28B7-APC antibodies (Table 3). Cells were then fixed in BD Stabilizing Fixative (BD Biosciences) and acquired on a BD FACSymphony A3 cytometer (BD Biosciences).

### 4.9. Quantification of HIV-Infected Cell Elimination

Human PBMCs were cultured in cRPMI with 5 µg/mL PHA for 72 h. PHA-stimulated PBMCs were infected with the vesicular stomatitis virus glycoprotein G (VSV-G) pseudotyped HIV-1 virus engineered with a green fluorescent protein (GFP) reporter (VSV-G/pNLG1-P2A-∆Env), as previously described [15]. Briefly, PBMCs were incubated with virus at an MOI of 0.4 for 4 h at 37 °C. After incubation, the PBMCs were washed three times with cRPMI by centrifugation at 200× *g* for 5 min. Infected PBMCs were then resuspended at 5 × 10^6^ cells/mL in complete medium with 10 U/mL IL-2 and incubated for 24 h before treatment with compounds. TACK and non-TACK compounds [16] were tested with or without 250 nM IDV. HIV-infected PBMCs were washed once, and either 100 nM TACK or non-TACK compound was added in four treatment conditions, including TACK alone, non-TACK alone, TACK with IDV, and non-TACK with IDV, then cultured for 72 h. The half maximal cytotoxic concentration (CC_50_) of both TACK and non-TACK compounds exceeded 40.5 µM. Moreover, cell viability under the aforementioned conditions, assessed using a ViCell XR Cell Viability Analyzer (Beckman Coulter, Brea, CA, USA), was greater than 92%. After incubation with compounds, cells were collected for measurement of cellular CA by CA-ICC and flow cytometry and viral RNA by qPCR with the methods described above. For the GFP-based microplate assay, cells were seeded in plates and analyzed with an Acumen X3 imager (SPT Labtech Ltd, Melbourn, UK) (488-nm laser) to count the number of GFP-positive cells. 

### 4.10. Data Analysis

Statistical analyses were performed on log-transformed data, and results were reported after transforming back to the original scale. The data were presented as the mean ± standard deviation. Graphs and figures were prepared using GraphPad Prism 10 (GraphPad Software, Inc., La Jolla, CA, USA). Statistical significance in group comparisons was denoted conventionally by *, *p* < 0.05 using the Student’s *t* test.

## Figures and Tables

**Table 1:** Proportion of CA-positive cells quantified by CA-ICC.

Cell Type	Antibody	Total Cells	CA-Positive Cells	CA-Positive Cells (%)
MOLT IIIB	mlgG	112,057	0	0.0
MOLT IIIB	HIV-CA	26,053	26,034	99.9
Jurkat	HIV-CA	69,410	0	0.0
Uninfected PBMCs	HIV-CA	145,907	0	0.0
In Vitro Infected PBMCs	HIV-CA	178,688	130,916	73.3

**Table 2:** CA-ICC coefficients of variation (CVs) for MOLT IIIB cells spiked into Jurkat cells.

CA-Positive Cells (%) *	Intra-Day (N = 3)	Inter-Day (N = 3)
Mean	7.33	7.07
SD	0.95	0.71
CV	12.97	10.08
Maximum	8.01	7.39
Minimum	6.24	6.23

* MOLT IIIB cells were spiked into Jurkat cells with a proportion of 7%.

**Table 3:** List of antibodies used in this work.

Reagents	Catalogue #	Manufacturer	City, State
mAb anti-HIV CA	HIV-018-48304	Capricorn	Murrieta, CA, USA
mAb anti-HIV CA	801136	ZeptoMetrix	Buffalo, NY, USA
mAb anti-CA 28B7-APC	MM-0289-APC	MediMabs	Montreal, QC, Canada
mAb anti-CA-KC57-PE	6604667	Beckman Coulter	Indianapolis, IN, USA
mAb anti-SIV p27	4324	ABL	Rockville, MD, USA
Mouse IgG isotype	31930	Thermo-Fisher	Carlsbad, CA, USA

## Data Availability

The data associated with the work are available upon request to the corresponding authors.

## Abbreviations

cART	Combination Antiretroviral Therapy
HIV	Human Immunodeficiency Virus
SIV	Simian Immunodeficiency Virus
CA	Capsid
ICC	Immunocytochemistry
mAb	Monoclonal antibody
MOI	Multiplicity of infection
PHA	Phytohemagglutinin
PBMCs	Peripheral Blood Mononuclear Cells
PLWH	People living with HIV
qPCR	Quantitative Polymerase Chain Reaction
IDV	Indinavir
PI	Protease Inhibitor
Simoa	Single Molecule Array
QVOA	Quantitative Viral Outgrowth Assay
GFP	Green Fluorescent Protein
IPDA	Intact Proviral DNA Assay
TACK	Targeted Activator of Cell Kill
